# Etranacogene dezaparvovec in people with hemophilia B and without adeno-associated virus serotype 5 neutralizing antibodies: a 4-year subgroup analysis of the Health Outcomes with Padua Gene; Evaluation in Hemophilia B (HOPE-B) trial

**DOI:** 10.1016/j.rpth.2025.103321

**Published:** 2025-12-30

**Authors:** Priyanka Raheja, Niamh O’Connell, Peter Verhamme, Peter Kampmann, Richard S. Lemons, Fei Wang, Sean Gill, Paul E. Monahan, Sandra Le Quellec, Frank W.G. Leebeek

**Affiliations:** 1 The Royal London Hospital Haemophilia Centre, Barts Health National Health Service Trust, London, United Kingdom; 2 National Coagulation Centre, St James's Hospital, Dublin, Ireland; 3 Department of Cardiovascular Medicine, University Hospitals Leuven, Leuven, Belgium; 4 Department of Haematology, Rigshospitalet, Copenhagen, Denmark; 5 Department of Pediatrics, University of Utah, Salt Lake City, Utah, USA; 6 CSL Behring, King of Prussia, Pennsylvania, USA; 7 Department of Haematology, Erasmus University Medical Center, Rotterdam, The Netherlands

**Keywords:** adeno-associated virus, factor IX, genetic therapy, hemophilia B, neutralizing antibody

## Abstract

**Background:**

In the phase 3 Health Outcomes with Padua Gene; Evaluation in Hemophilia B (HOPE-B) trial, a single dose of etranacogene dezaparvovec was administered to people with severe or moderately severe hemophilia B following a lead-in period (≥6 months) during which they received factor (F)IX prophylaxis. Participants were enrolled regardless of adeno-associated virus serotype 5 (AAV5)-neutralizing antibody (NAb) status at screening.

**Objectives:**

To determine efficacy, pharmacokinetic, and safety outcomes over 4 years postgene therapy in HOPE-B participants who were NAb-negative (NAb−).

**Methods:**

Participants provided serum samples for AAV5 NAb determination using an *in vitro* AAV5 transduction inhibition assay prior to etranacogene dezaparvovec infusion. Participants who were AAV5 NAb− at this time point were examined in the post hoc subgroup analysis.

**Results:**

In NAb− participants (*N* = 33), the mean adjusted annualized bleeding rate was significantly reduced between months 7 and 48 postetranacogene dezaparvovec vs lead-in (0.57 vs 3.80; *P* < .0001). In years 1 to 4, the annualized bleeding rates were 0.99, 0.72, 0.41, and 0.41, respectively (*P* < .0001 vs lead-in; *N* = 33 throughout). The mean (SD) endogenous FIX activity was 40.6 IU/dL (18.6) at month 6 postinfusion (*N* = 33), remained stable, and was 39.0 IU/dL (16.8) at year 4 (*N* = 33). Exogenous FIX consumption decreased by 99% during months 7 to 48 vs the lead-in period, and no NAb− participant returned to continuous FIX prophylaxis for 4 years postinfusion. No treatment-related oncogenic events or persistent late hepatotoxicity were observed.

**Conclusion:**

Etranacogene dezaparvovec proved to be highly effective, superior to FIX prophylaxis for bleeding protection, and safe for 4 years postinfusion in NAb− persons with severe or moderately severe hemophilia B.

## Essentials

• HOPE-B tested etranacogene dezaparvovec in people with hemophilia B with or without vector antibodies.
• This post hoc study reports 4-year results postgene therapy in antibody-negative patients.
• Etranacogene dezaparvovec was safe and reduced bleeding events compared with standard therapy.
• Around half of the patients produced stable FIX levels comparable to those of people without hemophilia.

## Graphical abstract

**Figure:**
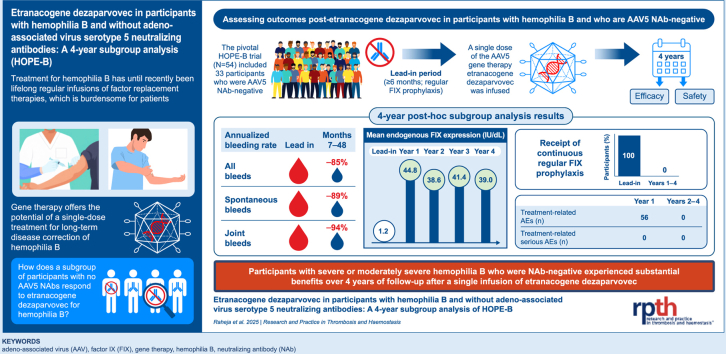

## 1 Introduction

Treatment for hemophilia B, an X-linked bleeding disorder resulting in deficient factor (F)IX activity, commonly involves FIX protein replacement therapies. Despite advances associated with newer FIX products, the lifelong need for regular infusions is burdensome for people with hemophilia B [1]. Infusion-related treatment burdens include the time required to prepare and administer treatment, pain during and/or after injections, and the need to store medication and supplies [2]. Such burdens may cause a delay in treatment, missed infusions, or a complete stop of prophylactic treatment, resulting in a deterioration of health outcomes for people with hemophilia B [3].

The recent development of gene therapy for hemophilia B offers the potential for a single-dose infusion, resulting in durable FIX expression, a substantial reduction in treatment burden, and improved patient quality of life [1,4]. The most common method for delivering the FIX coding sequence into cells uses adeno-associated virus (AAV), a nonreplicating single-stranded DNA parvovirus. AAVs offer several advantages for *in vivo* gene therapy, including the absence of known pathogenicity in humans. Wild-type AAVs demonstrate preferential tropism for specific target organs and typically persist as episomal circular DNA in the nucleus of host cells, with low genomic DNA integration rates [5]. However, when recombinant AAV vectors are used in clinical trials, dosing regimens may lead to higher absolute integration rates in targeted tissues, warranting careful consideration and long-term monitoring [6]. Several AAV serotypes, which differ in capsid amino acid sequence homology and other features, have been used in gene therapy [1,[7], [8], [9], [10]]. Wild-type AAVs occur naturally in the environment; therefore, people exposed to wild-type AAVs can develop neutralizing antibodies against the viral capsid [[11], [12], [13]] that cross-react with recombinant AAVs of the same or different serotypes; cross-neutralization has the potential to inhibit transduction of the target tissue during gene therapy [14]. Consequently, people with preexisting AAV-neutralizing antibodies have generally been excluded from clinical trials of AAV-based gene therapies. For example, a phase 3 gene therapy trial [15] that used a recombinant AAV serotype rh74 capsid excluded participants with AAV rh74-neutralizing antibodies; of 316 men screened, 188 (60%) were ineligible to enter the trial on this basis.

Etranacogene dezaparvovec is an AAV serotype 5 (AAV5)-based gene therapy with a codon-optimized gene expression cassette encoding the naturally occurring human FIX Padua (R338L) variant [16,17]. The primary analysis of the pivotal phase 3 Health Outcomes with Padua Gene; Evaluation in Hemophilia B (HOPE-B) trial (ClinicalTrials.gov identifier: NCT03569891) of etranacogene dezaparvovec (CSL222, HEMGENIX [CSL Behring]) demonstrated significantly improved bleeding outcomes in people with hemophilia B (FIX ≤ 2 IU/dL) who had previously been receiving standard-of-care continuous FIX prophylaxis [16,17]. Data from the previous phase 2b trial of etranacogene dezaparvovec showed that FIX expression was maintained for at least 5 years in participants [18]. In contrast to most AAV-based gene therapy clinical trials, HOPE-B enrolled participants with and without AAV5-neutralizing antibodies [17]. However, little data on long-term outcomes following gene therapy in persons with hemophilia B according to neutralizing antibody status are available. In the post hoc analysis of the HOPE-B study reported here, long-term efficacy and tolerability outcomes were assessed in the subgroup of participants without AAV5-neutralizing antibodies prior to etranacogene dezaparvovec infusion. Screening for AAV5-neutralizing antibody status is available not only in clinical trial settings but also in individuals with hemophilia B considering etranacogene dezaparvovec therapy in the real-world setting. Importantly, individuals without AAV5-neutralizing antibodies constitute the majority, representing approximately 55% to 60% of the global population [11,19,20].

This post hoc analysis provides the longest-term follow-up to date for a phase 3 study of systemically delivered, liver-directed AAV-based gene therapy for hemophilia B. By focusing on the most prevalent population, ie, individuals without AAV5-neutralizing antibodies, these results may inform clinical decision-making for these specific individuals and facilitate more accurate indirect comparisons with other gene therapy trials that restricted enrollment to antibody-negative patients.

## 2 Methods

### 2.1 Study participants

The HOPE-B study enrolled adult males with severe (FIX activity < 1 IU/dL) or moderately severe (FIX activity 1 to ≤2 IU/dL) hemophilia B. Participants were required to have been receiving stable, continuous FIX prophylaxis for ≥2 months prior to screening, with the specific dose and product determined by their physician. Informed consent was another inclusion criterion. Following screening, participants continued to receive their continuous FIX prophylaxis regimen throughout the lead-in period of 6 months or longer. Key exclusion criteria included a history of FIX inhibitors, active hepatitis B or C viral infection, and known severe infection or another significant concurrent uncontrolled medical condition. Neutralizing antibody positivity was not an exclusion criterion. Full eligibility criteria have been reported previously [17]. Participants eligible for this post hoc analysis of the HOPE-B study were AAV5-neutralizing antibody-negative on the day of dosing, prior to etranacogene dezaparvovec infusion.

### 2.2 Study design

HOPE-B was a phase 3, open-label, multinational study in which participants received a single intravenous dose of etranacogene dezaparvovec at 2 × 10^13^ genome copies/kg body weight and were planned to be followed for 5 years postgene therapy. The trial was conducted in accordance with the International Council for Harmonization Good Clinical Practice guidelines and the ethical principles stated in the Declaration of Helsinki. The study protocol was approved by independent ethics committees and institutional review boards at each study site.

The primary endpoint of HOPE-B was the annualized bleeding rate (ABR) during a 52-week period, from months 7 to 18 postgene therapy. Secondary endpoints included endogenous FIX activity (measured by a one-stage assay) at 26 and 52 weeks after steady-state FIX activity was reached, as well as factor replacement use, the frequency and severity of adverse events, and reactive corticosteroid use. We report here a post hoc subgroup analysis of efficacy, pharmacokinetic, and safety outcomes over 4 years in participants who were neutralizing antibody-negative on the day of dosing prior to etranacogene dezaparvovec infusion.

### 2.3 Analysis of AAV-neutralizing antibodies

Serum samples for AAV5-neutralizing antibody determination were obtained from participants during the screening period, lead-in period (at 8 and 4 weeks prior to etranacogene dezaparvovec infusion), and on the day of etranacogene dezaparvovec infusion. A central laboratory (Precision for Medicine) assessed AAV5-neutralizing antibody levels. This cell-based neutralizing antibody assay assessed the potential of participant serum to inhibit the *in vitro* transduction of mammalian cells with an AAV5 reporter vector expressing luciferase. Descriptions of the methodology for antibody determination have been reported previously [16,17].

### 2.4 Molecular analyses to assess neoplasm transformation

Molecular analyses were conducted by ProtaGene CGT GmbH, independent of the sponsor, to detect vector integration sites (IS) in DNA samples extracted from neoplastic tissue and blood. A detailed description of the analyses can be found in the Supplementary Methods.

Briefly, DNA was extracted using the QIAamp DNA Mini Kit (Qiagen). A polymerase chain reaction with vector-specific primers (hFIXco_FW and hFIXco_RV) was performed on 10 ng of DNA per sample, with vector-containing plasmids as a positive control. Whole-genome sequencing data were analyzed for IS detection and to perform somatic variant calling.

### 2.5 Statistical analysis

Demographic and baseline characteristics were summarized descriptively using sample size (*n*), mean, SD, minimum, maximum, median, and IQR for continuous measurements, and frequency and percentages (%) for categorical variables. Adjusted ABRs and comparisons of ABRs between the lead-in and postgene therapy periods were estimated using a repeated-measures regression model with a negative binomial distribution and generalized estimating equations, with an offset term to account for the paired design and the differential collection periods. One-stage activated partial thromboplastin time-based (SynthaSIL; Instrumentation Laboratory Co) FIX activity measurements (expressed as IU/dL) from the central laboratory were summarized descriptively. Postgene therapy FIX samples were considered contaminated and excluded from the analysis if drawn within 5 half-lives of FIX concentrate administration, based on the reported half-life of each product. Annualized FIX consumption, excluding FIX replacement for invasive procedures, was computed for each period by dividing total consumption by the time under observation (in years) and compared between the postgene therapy phase and lead-in phase using a 2-sided paired *t*-test (using the pair of values for each participant). The analyses reported here describe a retrospective, post hoc examination of prospectively collected data from the lead-in phase and 4 years of follow-up for all participants treated in the HOPE-B phase 3 trial, which was not specifically powered to detect significant differences or associations. All analyses were performed in SAS 9.4 (SAS Institute); figures were generated using GraphPad.

## 3 Results

### 3.1 Participants

The study began on June 27, 2018, and this 4-year post hoc analysis includes data through June 3, 2024. Overall, 33 of 54 participants in HOPE-B were AAV5-neutralizing antibody-negative on the day of dosing, prior to etranacogene dezaparvovec infusion. Baseline demographics of these participants are shown in the Table. Most participants (85%) had a severe hemophilia B diagnosis (FIX < 1 IU/dL), and around half (52%) had experienced a prior hepatitis C viral infection. All 33 participants completed 4 years of follow-up after etranacogene dezaparvovec infusion.

**Table:** Baseline demographics and clinical characteristics.

Characteristic	Neutralizing antibody-negative participants (*N* = 33)
Age (y), mean (SD; min-max)	39.5 (14.5; 21-73)
Race/ethnicity, *n* (%)	
White	28 (85)
Hispanic or Latino	2 (6)
Other	1 (3)
Missing	2 (6)
Positive HIV status, *n* (%)	2 (6)
Prior hepatitis B, *n* (%)	4 (12)
Prior or ongoing hepatitis C^a^, *n* (%)	17 (52)
Severity of hemophilia B at diagnosis, *n* (%)	
Severe (FIX < 1 IU/dL)	28 (85)
Moderately severe (FIX ≥ 1 IU/dL and ≤ 2 IU/dL)	5 (15)
Prescreening FIX treatment, *n* (%)	
Extended half-life	17 (52)
Standard half-life	16 (48)
Participants with zero reported bleeds during the lead-in period, *n* (%)	11 (33)

FIX, factor IX; max, maximum; min, minimum.

a Most participants had experienced prior hepatitis C infections (*n* = 16); 1 participant was undergoing eradication of hepatitis C at the time of screening and had evidence of hepatitis C virus eradication at the time of etranacogene dezaparvovec infusion.

### 3.2 ABRs

**Figure 1:**
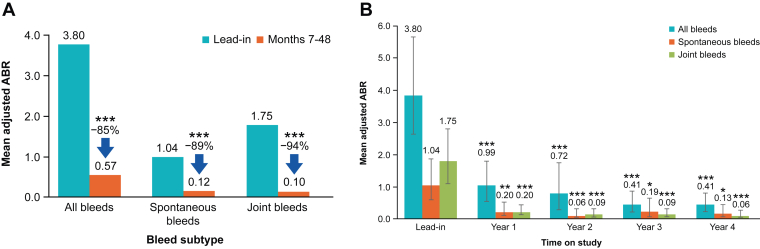
Comparison of annualized bleeding rates (ABRs) between (A) lead-in and months 7 to 48 and (B) lead-in and years 1 to 4 postgene therapy (*N* = 33). ∗*P* < .01 vs lead-in; ∗∗*P* < .001 vs lead-in; ∗∗∗*P* < .0001 vs lead-in. Error bars in B show the 95% CI.

In neutralizing antibody-negative participants (*N* = 33), the mean adjusted ABR (all bleeds) was reduced by 85% (2-sided Wald 95% CI, 75%-91%; *P* < .0001), from 3.80 during lead-in to 0.57 during months 7 to 48 postetranacogene dezaparvovec infusion (Figure 1A). The mean adjusted ABRs for all bleeds in years 1 to 4 postetranacogene dezaparvovec infusion were 0.99, 0.72, 0.41, and 0.41, respectively (all *P* < .0001 vs lead-in; *N* = 33 at all time points; Figure 1B).

Compared with the lead-in period, the mean adjusted ABR for spontaneous bleeds was reduced by 89% (2-sided Wald 95% CI, 64%-96%; *P* < .0001), from 1.04 during lead-in to 0.12 during months 7 to 48 (Figure 1A). The mean adjusted spontaneous ABRs were 0.20, 0.06, 0.19, and 0.13 in years 1 to 4 postetranacogene dezaparvovec infusion, respectively (*P* < .001, *P* < .0001, *P* < .01, and *P* < .01, respectively, vs lead-in; Figure 1B).

Similarly, the mean adjusted ABR for joint bleeds was reduced by 94% (2-sided Wald 95% CI, 88%-97%; *P* < .0001), from 1.75 during lead-in to 0.10 during months 7 to 48 (Figure 1A). In years 1 to 4, the mean adjusted joint ABRs were 0.20, 0.09, 0.09, and 0.06 postetranacogene dezaparvovec infusion, respectively (all *P* < .0001 vs lead-in; Figure 1B).

These reductions in ABR and the associated *P* values met the statistical thresholds for both noninferiority and superiority compared with the lead-in standard-of-care treatment.

### 3.3 Endogenous FIX activity

**Figure 2:**
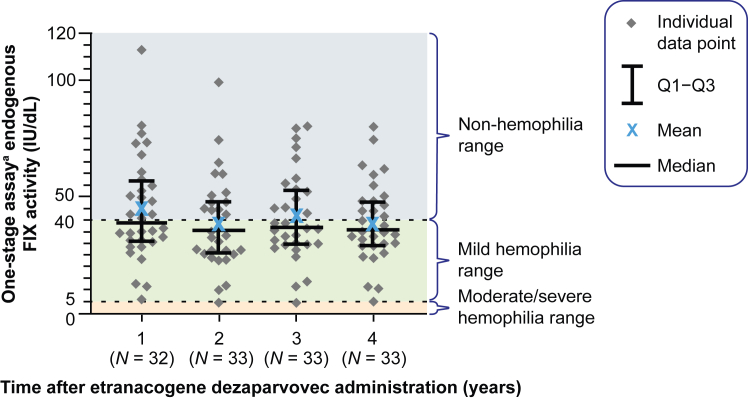
Endogenous factor (F)IX activity levels for years 1 to 4 posttreatment (*N* = 33). ^a^Assessed by one-stage activated partial thromboplastin time FIX activity assay. Only uncontaminated samples were included in this analysis (ie, blood sampling did not occur within 5 half-lives of exogenous FIX use). Q1-Q3, IQR.

All 33 neutralizing antibody-negative participants expressed endogenous transgene-derived FIX postgene therapy (Figure 2). The mean (SD) endogenous FIX activity was 40.6 IU/dL (18.6) in month 6 (*N* = 33), remained stable for 4 years postgene therapy, and was 39.0 IU/dL (16.8) in year 4 (*N* = 33). The median (range) FIX activity in year 4 was 35.7 IU/dL (4.7-80.1).

### 3.4 Use of exogenous FIX

**Figure 3:**
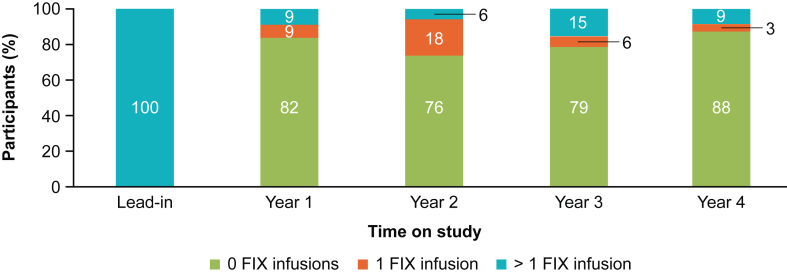
Proportion of neutralizing antibody-negative participants who required exogenous factor (F)IX infusions by year (*N* = 33). FIX infusions for the management of invasive procedures were excluded from this analysis.

Over the 4-year time period reported here, no neutralizing antibody-negative participant returned to continuous exogenous FIX prophylaxis following etranacogene dezaparvovec infusion. In each year postgene therapy, approximately 80% of neutralizing antibody-negative participants did not require any exogenous FIX infusions (Figure 3). During the lead-in period, bleeds requiring FIX treatment accounted for 82% of total bleeds; postgene therapy, 37% of all bleeds over 4 years required exogenous FIX treatment. Exogenous FIX consumption, excluding invasive procedures, decreased by 99%, from a mean (SD) of 264,888 IU/y (153,545) during the lead-in period to a mean (SD) of 1878 IU/y (3337) during months 7 to 48 postgene therapy (mean [SE] reduction of 263,010 IU/y [26,615]; *P* < .0001; *N* = 33).

### 3.5 Safety

**Figure 4:**
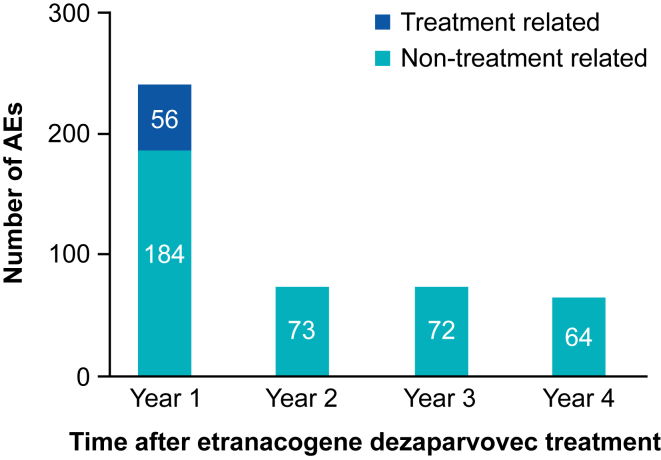
Number of treatment-related and non–treatment-related adverse events (AEs) by year postgene therapy (*N* = 33).

Of 455 treatment-emergent adverse events reported in neutralizing antibody-negative participants (Figure 4) during years 1 to 4 postetranacogene dezaparvovec infusion, 78% were mild, 19% moderate, and 3% severe. Overall, 22 of 33 participants experienced treatment-related adverse events during the first 3 months following etranacogene dezaparvovec infusion; no treatment-related adverse events were reported during months 3 to 42 of follow-up, and 1 participant reported 3 treatment-related adverse events during months 43 to 48. The most frequent treatment-related adverse event was transient alanine transaminase elevation in 6 (18%) participants (Supplementary Figure S1). These elevations occurred between days 22 and 71 postetranacogene dezaparvovec infusion. The peak alanine aminotransferase level for 1 participant was 2-fold the upper limit of normal, for 3 participants, peak alanine aminotransferase elevations were 1- to 2-fold the upper limit of normal, while for 2 participants, peak alanine aminotransferase elevations were approximately 2-fold the value of the participants’ pregene therapy baseline alanine aminotransferase levels; however, these elevations remained within normal limits. Five of 6 participants with treatment-related alanine aminotransferase elevations and 1 with non–treatment-related alanine aminotransferase elevations received a reactive course of corticosteroid therapy, with a mean (SD) total duration of corticosteroid use of 79.5 days (30.3). Time to receipt of corticosteroid treatment following alanine aminotransferase elevation ranged from 0 to 21 days. The mean (SD) endogenous FIX activity at or near the time of corticosteroid initiation was 20.8 IU/dL (10.3; *n* = 6). The mean (SD) endogenous FIX activity remained stable for 4 years postgene therapy, with a value of 20.5 IU/dL (13.5; *n* = 6) in year 4. The median (range) FIX activity in year 4 was 18.5 IU/dL (4.7-37.6; *n* = 6; Supplementary Figure S1).

No persistent late hepatotoxicity was observed, including participants who experienced early liver inflammation and those with a history of chronic viral hepatitis. No serious adverse events considered related to treatment, the development of inhibitors, or thrombotic events were reported. No oncogenic events considered related to treatment were reported. During year 4, 1 serious adverse event, a glossopharyngeal schwannoma, was observed in a neutralizing antibody-negative participant and was investigated by molecular analysis for vector integration. No evidence of AAV5 vector DNA in tumor or control samples was detected by polymerase chain reaction; no integration events were identified in affected tissue using whole-genome sequencing, while premalignant signatures of somatic *NF2* defects, consistent with the development of a schwannoma, were found; consequently, this serious adverse event was considered unrelated to treatment. A detailed description of the patient narrative, molecular analyses of IS detection, and identification of relevant genetic signatures and corresponding findings are provided in Supplementary Results as well as Supplementary Figures S2 and S3.

## 4 Discussion

The present study provides 4-year follow up data from a phase 3 gene therapy trial which support the sustained efficacy and durability of AAV-based gene therapy for hemophilia B. Additionally, the post hoc analysis includes detailed outcomes for the participants who tested negative for AAV5-neutralizing antibodies prior to etranacogene dezaparvovec. This subgroup not only represents the largest subset of the trial but also reflects the expectation that most individuals with hemophilia B do not have preexisting neutralizing antibodies to AAV5, in contrast to other AAV serotypes [11,19,20].

Neutralizing antibody-negative participants demonstrated stable endogenous FIX activity over 4 years of follow-up postgene therapy, accompanied by durable bleed protection and limited treatment-related adverse events, with none reported after month 3. While it has been previously reported that 2 participants with preexisting AAV5-neutralizing antibodies did not express endogenous transgene-derived FIX Padua protein following treatment with etranacogene dezaparvovec [16,17], all treated neutralizing antibody-negative participants expressed stable endogenous transgene-derived FIX throughout the 4-year analysis period, with a median value of one-stage FIX activity levels at 4 years of follow-up of 35.7 IU/dL; the median value for the intention-to-treat population (*N* = 54), ie, including neutralizing antibody-positive participants, at 4 years was 34.6 IU/dL (data not shown). Approximately half of the neutralizing antibody-negative participants had endogenous FIX activity within the nonhemophilia range. However, the response was variable, with endogenous FIX values ranging from 4.7 to 80.1 IU/dL in year 4. The impact of early alanine aminotransferase elevation on FIX expression was one important contributor to the observed wide variation in response; 3 participants with alanine aminotransferase elevations also had the lowest endogenous FIX values (<15 IU/dL) in years 1 to 4. Of note, these participants already had the lowest FIX expression prior to the occurrence of alanine aminotransferase elevation. With the exception of these 3 individuals, all neutralizing antibody-negative participants maintained a FIX activity > 20 IU/dL from months 7 to 48. Transient liver function abnormalities treated with corticosteroids during the first 6 months after gene therapy were not associated with subsequent instability or decreases in endogenous FIX activity at months 7 to 48 of follow-up; FIX expression that was preserved at discontinuation of corticosteroids was, in general, maintained at stable levels for the remainder of the follow-up period. Moreover, all endogenous FIX levels, including the lower values, allowed discontinuation of continuous FIX prophylaxis in the first weeks after gene therapy, and all neutralizing antibody-negative participants remained free of continuous FIX prophylaxis throughout the 4-year analysis period.

Focusing exclusively on the subset of HOPE-B participants with undetectable preexisting AAV5-neutralizing antibodies is valuable, as it enables a meaningful indirect comparison with other AAV-based gene therapy trials for both hemophilia A and hemophilia B. This is because most of these trials [21] have excluded participants who were baseline AAV antibody-positive to their respective AAV vectors, primarily due to concerns that AAV-neutralizing antibodies would prevent transduction of target cells.

Moreover, few studies have reported long-term pharmacokinetic and efficacy data on hemophilia gene therapies. Long-term data are essential for determining the durability and safety of this recently developed therapeutic modality and for guiding the development of future gene therapies. Long-term maintenance of therapeutic levels of endogenous FVIII expression has been challenging in gene therapy trials for people with hemophilia A [12,22]. In a 5-year analysis of the phase 3 GENEr8-1 FVIII gene therapy trial of valoctocogene roxaparvovec (*N* = 134; all of whom were AAV5 immunoglobulin G-binding antibody-negative pregene therapy), the mean (SE) chromogenic assay-assessed endogenous FVIII activity was 13.7 IU/dL (2.1; mean one-stage assay-assessed FVIII: 24.0 IU/dL) at year 5; the mean ABR for treated bleeds was 0.6, and 78% of patients had zero bleeds in year 5 [23]. However, despite these relatively positive outcomes, 19% of patients required reinitiation of FVIII replacement treatment within 5 years postgene therapy [22,23].

Using the gain-of-function Padua FIX variant in hemophilia B gene therapy, long-term stable expression of FIX at protective levels has been observed, although the extent of protection appears to vary according to gene therapy [15]. Three- and 5-year follow-up data from the initial phase 2b trial of etranacogene dezaparvovec (*n* = 3; all participants were neutralizing antibody-positive) also showed sustained endogenous FIX activity (36.9 and 45.7 IU/dL at years 3 and 5 postgene therapy, respectively), a significant reduction in bleeding events, and a significant decrease in requirement for exogenous FIX, supporting the longer-term therapeutic benefit of FIX Padua-based gene therapy [18,24]. The HOPE-B post hoc analysis reported herein found a mean adjusted ABR for all bleeds of 0.57 during months 7 to 48 postetranacogene dezaparvovec infusion, and importantly, showed superior results compared with other vectors in late-stage development. Indeed, another phase 3 study (*N* = 45) of hemophilia B gene therapy with fidanacogene elaparvovec, also utilizing FIX Padua, reported a mean endogenous one-stage FIX activity of 26.9 IU/dL at month 15 postgene therapy in people with hemophilia B, all of whom were AAV-neutralizing antibody-negative (for the AAV rh74 serotype used in that trial) [15]. This resulted in an ABR for all bleeds of 1.28 at month 15 postgene therapy. Although longer-term follow-up of this trial is not yet available, it is notable that 6 of 45 participants have already returned to continuous FIX prophylaxis within fewer than 15 months after fidanacogene elaparvovec administration. In contrast, it is remarkable that none of the neutralizing antibody-negative participants returned to continuous FIX prophylaxis during the 4-year period postetranacogene dezaparvovec reported here. The mechanisms underlying the observed superior outcomes with etranacogene dezaparvovec in neutralizing antibody-negative participants, compared with other AAV vectors, remain unknown. A range of factors, including capsid-specific immune responses, transduction efficiency and dosing, vector genome attributes (such as CpG content), manufacturing process, and the recipient’s hepatic function and immunological profile, can collectively influence both the durability and extent of transgene expression, as well as the potential for related hepatotoxicity. Adverse events considered related to etranacogene dezaparvovec, which was administered at a dose of 2 × 10^13^ genome copies/kg body weight, occurred in 67% of neutralizing antibody-negative participants, all within the first 3 months after gene therapy administration. The most common treatment-related adverse event was transiently increased alanine aminotransferase levels, occurring in 18% of participants, which were successfully managed with corticosteroid therapy, and stable endogenous FIX activity was achieved; none of these participants required a return to continuous exogenous FIX prophylaxis. However, due to potential effects on hepatocyte-derived FIX Padua expression, as previously discussed, it is important to closely monitor liver transaminases in the first few months after gene delivery. This allows for immediate supportive care with corticosteroids to minimize the impact on treatment efficacy.

In contrast, in BENEGENE-2 [15], a phase 3 trial in which participants received a single dose of fidanacogene elaparvovec at 5 × 10^11^ genome copies/kg body weight, 24/45 (53%) participants experienced increased transaminase levels. Of the 6 participants who resumed continuous exogenous FIX prophylaxis due to low FIX activity, all had received at least 1 course of glucocorticoids (2 of these participants received 2 courses of steroids due to increased transaminase levels). A recent phase 1 study (*N* = 10) of BBM-H901, an AAV vector expressing Padua FIX, reported 1 (10%) participant with treatment-related alanine aminotransferase elevation, which was associated with a decrease in FIX activity [25]. In this study, participants were excluded if they had a hepatitis B or C virus infection, alanine aminotransferase levels higher than 2-fold the upper limit of normal, or liver conditions such as liver fibrosis stage ≥3; all participants received per-protocol prophylactic glucocorticoids from day 7 prior to BBM-H901 infusion and for approximately 7 to 9 weeks afterward [25]. This suggests that prophylactic corticosteroids neither fully prevent postgene therapy elevations of alanine aminotransferase nor support long-term stability of FIX activity. Notably, etranacogene dezaparvovec was associated with infrequent and mild alanine aminotransferase elevations, all effectively managed with short, reactive corticosteroid courses, and was not associated with decreases in FIX levels after support with corticosteroids was initiated, underscoring that timely corticosteroid initiation at the first sign of alanine aminotransferase elevation is essential for maintaining stable FIX levels.

Regarding long-term safety, the case of the participant who developed a schwannoma was comprehensively evaluated through molecular analyses, including tests of vector integration. There was no evidence of vector DNA in the tissues analyzed, and no vector integration was detected in the schwannoma sample, thereby excluding vector involvement. The results were consistent with the established preferential hepatic tropism of the AAV5 serotype and aligned with the low integration frequency characteristic of recombinant AAV vectors, as described in previous clinical reports [26,27]. After more than 2 decades of clinical use, AAV-based gene therapy for hemophilia has not resulted in any confirmed cases of AAV-related cancer, despite concerns about potential insertional mutagenesis [22]. While ongoing long-term follow-up studies continue to characterize the safety profile and address any latent risks, current evidence increasingly supports the benign nature of AAV vector integration in the clinical setting. However, an estimated 0.1% to 3% of liver-targeted recombinant AAV vectors may integrate into hepatocyte DNA, potentially corresponding to many millions of hepatic integration events at a dose of 2 × 10^13^ genome copies/kg body weight. Therefore, continued monitoring, with particular focus on hepatic neoplasms, including long-term registry follow-up, remains scientifically valuable, especially to reinforce confidence in the safety of AAV-based therapies and to guide evidence-based risk-benefit assessments and postmarketing strategies [28].

Limitations of this post hoc subgroup analysis include the fact that it was not prespecified and the relatively small sample size. However, given the low prevalence of people with hemophilia B in the general population, we believe the insights into the efficacy and safety of etranacogene dezaparvovec in neutralizing antibody-negative participants generated by this analysis have high clinical value.

## 5 Conclusions

All persons with severe to moderately severe hemophilia B in the HOPE-B trial who tested negative for AAV5-neutralizing antibodies prior to etranacogene dezaparvovec infusion expressed endogenous FIX at therapeutic levels and achieved durable bleed protection over a 4-year period, with no participants returning to continuous FIX prophylaxis during this time frame. No treatment-related adverse events occurred after the first 3 months following gene therapy; importantly, no events of AAV5-associated genotoxicity or persistent late hepatotoxicity were observed. These data provide important information that will help physicians and individuals with hemophilia B considering etranacogene dezaparvovec gene therapy assess and understand potential outcomes, enabling informed decision-making.
