# Supplementary material for: Etranacogene dezaparvovec in people with hemophilia B and without adeno-associated virus serotype 5 neutralizing antibodies: a 4-year subgroup analysis of the Health Outcomes with Padua Gene; Evaluation in Hemophilia B (HOPE-B) trial
Source: Res Pract Thromb Haemost. 2025 Dec 30;10(1):103321. doi: 10.1016/j.rpth.2025.103321 (PMC12857339; doi:10.1016/j.rpth.2025.103321)

# **Supplementary materials**

## ***Supplementary methods***

Molecular analyses were conducted by ProtaGene CGT GmbH (Heidelberg, Germany) independently from the sponsor.

### *1.1 DNA isolation*

DNA was extracted from tissue and blood using the QIAamp DNA Mini Kit (Qiagen) following the manufacturer’s instructions. Approximately 25 mg brain tissue was homogenized using a TissueLyser device (Qiagen) followed by digestion overnight with proteinase K. For blood samples, PBS was added to the sample followed by 10 min digestion with proteinase K. Subsequently, genomic DNA was extracted using spin-columns, eluted in H_2_O, and stored at −20°C.

### *1.2 Test for presence of vector*

To detect if a sample contained vector DNA, a polymerase chain reaction (PCR) with vector-specific primers (hFIXco_FW and hFIXco_RV) was performed. Briefly, 10 ng DNA per sample were taken as a template. The PCR amplicons obtained were assessed using automated electrophoresis (TapeStation, Agilent Technologies). Vector-containing plasmid was used as a positive control.

### *1.3 WGS library preparation*

Whole genome sequencing (WGS) library preparation was performed using the TruSeq DNA PCR-free library preparation kit (Illumina). Briefly, 1 µg genomic DNA per sample was used as the input and sheared to a median length of 350 bp using the Covaris M220 instrument according to the manufacturer’s instructions. This process was repeated to generate sufficient library material. Libraries were sequenced using 150bp paired-end sequencing on a NovaSeq 6000 device (Illumina).

### *1.4 WGS integration site analysis and variant calling*

WGS sequencing data were analyzed for the detection of integration sites (IS) and to perform somatic and germline variant calling. Briefly, raw sequencing reads were processed for initial alignment with the provided adeno-associated virus (AAV) vector reference sequence (pVD1065) using the BWA-MEM aligner (Li 2013). Subsequently, reads with vector signatures were extracted, and this subset of data was processed for IS analysis with human and vector reference genome (hg38 and vector reference) using the GENE-IS tool suite (Afzal et al. 2017). Variant calling was done using the Illumina BaseSpace platform (www.illumina.com). Two standard workflows, DRAGEN Somatic and Germline were employed, as designed by Illumina. These included rapid alignment, followed by variant calling. Standard filters were applied, including ‘PASS’ to remove likely false positives and “lowComplexityRegion” to exclude variants in repetitive regions and finalized using annotation tools, e.g., COSMIC and Ensembl. Somatic variants were called using the DRAGEN Somatic pipeline. Variants passing these criteria were retained for downstream analysis. To filter further, only variants present in established databases (COSMIC, HGNC, Ensembl, ClinVar) and predicted to have functional impact were considered for interpretation. Germline variants in glossopharyngeal schwannoma and matched normal sample were called using the DRAGEN Germline pipeline. For germline small nucleotide variants (SNVs), only variants annotated in HGNC and Ensembl, with predicted functional impact, genotype quality >20, and supported by ≥10 reads were retained. Variants with population frequency >1% were excluded. For structural variants (SVs) and copy number variants (CNVs), COSMIC gene fusions in BaseSpace were used for annotation. Additional criteria included paired-end read count >5, split-read support >5–10 per side, and confidence interval <20.

## ***Supplementary Results***

### *2.2. Participant with glossopharyngeal schwannoma*

The participant was a 61-year-old (at the time of study screening) male with severe hemophilia B (FIX activity at screening <1IU/dL). Past history included hepatitis C infection for approximately 22 years which was documented as resolved five years prior to enrolment in HOPE-B trial.

Approximately three years posttreatment with full dose etranacogene dezaparvovec (day of etranacogene dezaparvovec administration = study day 0, D0), the participant presented with bilateral progressive hearing loss and intermittent lightheadedness or imbalance. No other neurological symptoms were noted.

A magnetic resonance imagining (MRI) brain scan on (study D1367) showed a 3.7 x 2.8 cm multicystic mass within the right cerebellopontine angle, which most likely arose intra axially, although there was a possibility that the lesion arose anterior to the cerebellum. On study D1368, a computerized tomography (CT) scan of the neck and thorax/abdomen/pelvis confirmed a 3.9 x 2.8 x 2.9 cm (transverse x craniocaudal x anterior-posterior) multicystic mass within the right cerebellopontine angle, with no primary tumor or metastasis and borderline enlarged inguinal lymph nodes laterally.

#### 2.2.1 Surgery and pathology

On study D1385, subtotal debulking of the cerebellopontine angle mass was performed. Exogenous FIX was used for the management of the surgery (5500 IU of ALPROLIX® on pre-surgery and a total of 8000 IU of ALPROLIX across 10 days post-surgery). No intraoperative or post-operative bleeds were recorded, no thrombotic events occurred, and no FIX inhibitor developed.

Histopathological analysis of the mass found a benign glossopharyngeal schwannoma, WHO grade 1.

#### 2.2.2 AAV integration studies and molecular characterization

To investigate the etiology of glossopharyngeal schwannoma focusing on a potential involvement of etranacogene dezaparvovec vector integration into the host genome during neoplastic transformation, the sponsor coordinated with a pathologist at the surgical center for collection, processing, and shipment of brain tumor tissue for molecular analysis at Protagene CGT GmbH (Heidelberg, Germany). Normal CNS tissue was not available as a non-tumor control; therefore a whole blood sample was used.

First, a semi-quantitative PCR was carried out using vector-specific primers located at the 3’-end of the transgene to test for the presence of vector DNA. No vector signal was detected in blood or tumor samples. Despite the absence of vector signal, additional in-depth analysis was done: WGS was applied for identifying somatic variants that had the potential to explain the tumor origin. Additionally, germline variants and the ploidy and purity of the tumor were investigated. WGS data were also assessed for the detection of vector integration sites (IS).

##### 2.2.2.1 *WGS analysis - Somatic and germline variant detection*

The raw reads for both samples provided passed quality control. The average base quality was 36 and the average coverage was estimated to 135 or 181 for brain tumor or non-tumor control, respectively. The purity of the tumor sample was estimated to be 76%. The estimated chromosome count was 45.03 and the estimated ploidy was 1.93.

The tumor sample showed partial loss of chromosome 22. Analysis of the B allele frequency revealed values clustering near 0.0 and 1.0, with a significant reduction around the 0.5 mark, consistent with a loss of heterozygosity. 35 of 51 million base pairs on chromosome 22 (region 15,166,285 to 50,780,639) were lost in one copy, which is roughly the entire distal end of chromosome 22q (**Supplementary Figure S2**). The estimated chromosome counts and ploidy match with the partial loss of chromosome 22.

A total of 545 genes are located in the deleted region of chromosome 22, including *NF2*, a well-known tumor suppressor. Mutations in the *NF2* gene on chromosome 22q12.2 are the most common genetic cause of schwannomas (Kehrer-Sawatzki et al., 2017; Pathmanaban et al., 2017; Gao et al., 2020). The *NF2* gene encodes the merlin protein, which regulates cell proliferation and motility (Kehrer-Sawatzki et al., 2017). Patients with schwannoma frequently harbor typical truncating mutations of the *NF2* gene and loss of heterozygosity of the surrounding region of chromosome 22 (Jacoby et al., 1997; Gao et al., 2020).

Two additional genes on chromosome 22, *LZTR1* and *SMARCB1*, have been studied in schwannoma cases (Dhamija et al., 1993). Somatic *LZTR1* pathogenic variants have been identified in about one fifth of glioblastomas (Frattini et al., 2013). A somatic variant in the gene *SMARCB1* has been detected in sporadic meningiomas occurring as single tumors in the absence of any other findings of schwannomatosis (Schmitz et al., 2001). Notably, *NF2*, *LZTR1*, and *SMARCB1* are all absent in one copy of the chromosome 22.

SNV analysis retained 50 variants after applying the filters, among those a 29bp deletion in the second copy of *NF2* gene. The deletion was reported in COSMIC data and has been published (Clark et al., 2013). The variant affects the coding region, leading to a deletion of 5 nucleotides and a change at protein level (c.336_340; p.E112Dfs*16) (**Supplementary Figure S3**). As the gene *NF2* was absent in one copy and the protein was affected in the other copy, the tumor sample genome effectively lacks a functional copy of the *NF2* gene.

Furthermore, germline single nucleotide variants were analyzed; two *NF2* and four *LZTR1* germline SNVs were identified. No variants were identified in *SMARCB1*. Although these specific variants have not been directly linked to schwannoma, published studies strongly implicate *NF2* and *LZTR1* germline variants in schwannoma predisposition (Li et al., 2022; Schmidt et al., 2001), suggesting that the variants identified here may contribute to disease development.

##### *3.2.2.2 IS analysis by WGS*

WGS data from brain tumor and peripheral blood were processed for IS retrieval with human reference genome (hg38) and the provided pVD1065 vector sequence. No IS were detected in either sample. Extracted DNA from the CNS tumor sample was limited, WGS analysis was prioritized over targeted AAV integration site analysis by TES.

#### 3.2.3 Summary with interpretation

Taken together, the molecular analysis detected clinically relevant somatic variants in the *NF2* gene. In contrast, no vector IS could be detected by WGS, indicating that the tumor origin was most likely not related to etranacogene dezaparvovec.

# ***Figures***

**Supplementary Figure S1**. Change in endogenous factor IX activity over 48 months in individual participants (n=33)


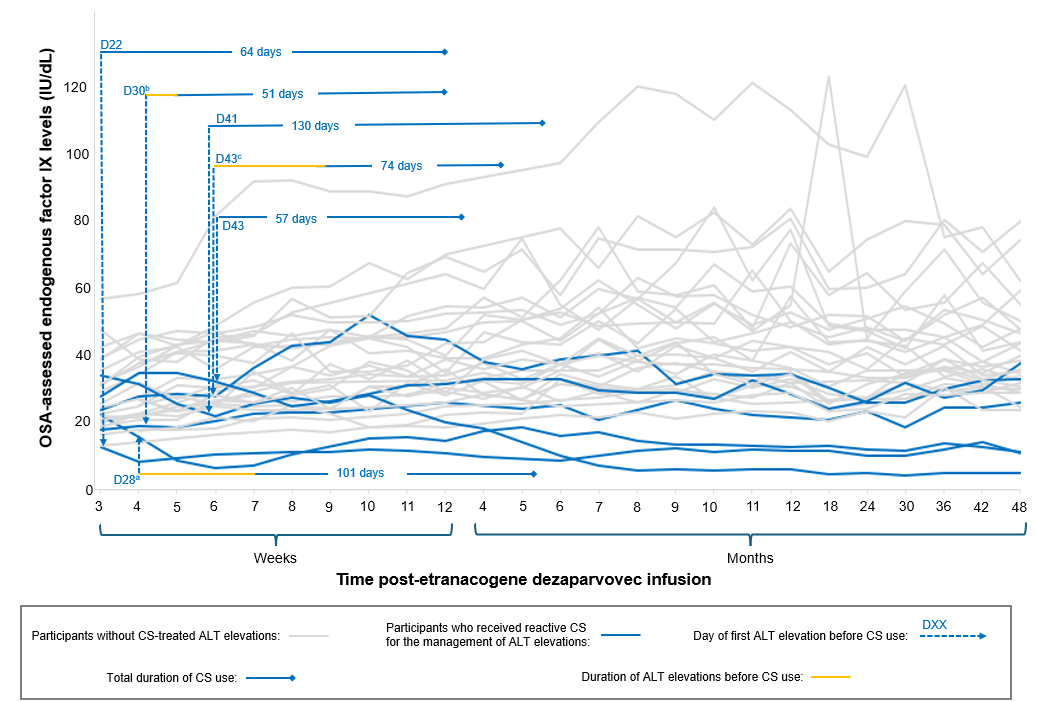


Uncontaminated samples were included in this analysis (i.e., blood sampling did not occur within 5 half-lives of exogenous factor IX use).

^a^Corticosteroids administered 21 days following day of first ALT elevation; ^b^Corticosteroids administered 6 days following day of first ALT elevation; ^c^Corticosteroids administered 18 days following day of first ALT elevation.
ALT, alanine aminotransferase; CS, corticosteroids; DXX, day of first ALT elevation before CS use; OSA, one-stage activated partial thromboplastin time factor IX activity assay.

**Supplementary Figure S2**. Chromosome 22 arm p and q representation. Red boxed area represents the lost part in one copy of the chromosome (q arm).

**
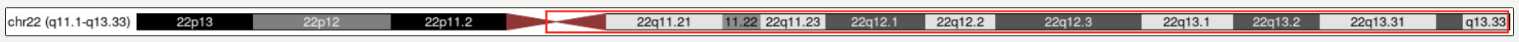
**

**Supplementary Figure S3***. NF2* somatic variant**.** The panel shows tumor (bottom) vs normal sample (top). The SNV starting from position 29,639,189 to position 29,639,218 on chromosome 22 is depicted by parallel lines in grey. The deletion frameshift leads to a change at the protein level from the glutamic acid (E) at position 112 to an aspartic acid (D).


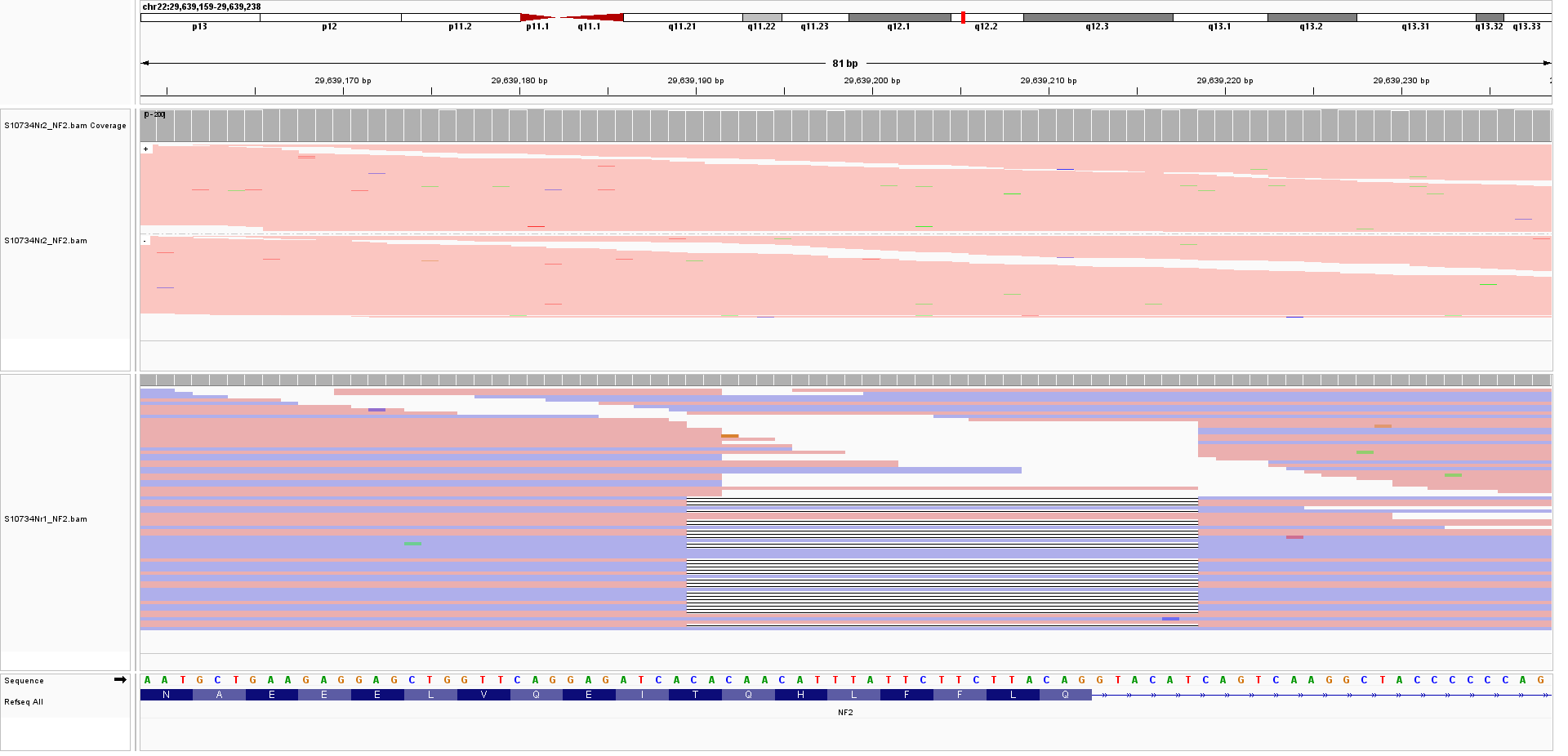

Supplement: Supplementary Methods [file mmc1.docx]
